# Prolonged febrile illness due to CTX-M-15 extended-spectrum β-lactamase-producing *Klebsiella pneumoniae* infection in Nigeria

**DOI:** 10.4102/ajlm.v1i1.16

**Published:** 2012-06-04

**Authors:** Oladipo A. Aboderin, Olufemi Adefehinti, Babatunde W. Odetoyin, Amadin A. Olotu, Iruka N. Okeke, Olugbenga O. Adeodu

**Affiliations:** 1 Department of Medical Microbiology and Parasitology, Obafemi Awolowo University, Ile-Ife, Nigeria; 2 Department of Medical Microbiology and Parasitology, Obafemi Awolowo University Teaching Hospitals Complex, Ile-Ife, Nigeria; 3 Department of Paediatrics, Obafemi Awolowo University Teaching Hospitals Complex, Ile-Ife, Nigeria; 4 Department of Biology, Haverford College, Haverford, United States; 5 Department of Paediatrics and Child Health, Obafemi Awolowo University, Ile-Ife, Nigeria

## Abstract

We report on an 8-year-old patient with septicaemia unresponsive to therapy for five weeks. Undetected, extended-spectrum β-lactamase (ESBL) production by the infecting *Klebsiella* strain was regarded as responsible for treatment failure. Intravenously administered imipenem during the sixth week led to sustained resolution of fever. Resource-limited hospitals can incur prohibitive costs from ESBL-producer infections because of diagnostic limitations and consequent treatment failure involving prolonged supportive therapy.

## Introduction

The first report of plasmid-encoded β-lactamase capable of hydrolysing the extended-spectrum cephalosporins was published in 1983.^1^ Since then, extended-spectrum β-lactamases (ESBLs) have become an increasingly important resistance mechanism among Enterobacteriaceae worldwide. A 2006 report of the Infectious Diseases Society of America listed ESBL-producing *Klebsiella pneumoniae* and *Escherichia coli* among drug-resistant microbes for which new therapies are urgently needed.^2^ Reports show that the ESBL problem is rapidly evolving and increasing in severity and scope with the discovery of new ESBLs, particularly the CTX-M types, which have become the most prevalent.^3^ The β-lactamases are the greatest threat to the usefulness of β-lactam antibiotics such as the penicillins and cephalosporins. Of all the different types of β-lactamases, ESBLs currently have the greatest clinical impact in terms of diversity and distribution as well as the ability to hydrolyse expanded-spectrum third generation cephalosporins. The earliest variants of ESBLs originated as a result of point mutations in the genes for broad-spectrum β-lactamases whilst newer ones, including the most successful such as CTX-M, arose by acquisition from the environmental metagenome through horizontal gene transfer.^4^ Cephalosporins as bactericidal, cell wall-active β-lactam agents were introduced in the 1980s and as a result of effectiveness against broad-spectrum β-lactamases became standard for treatment of severe conditions such as bloodstream infections, pneumonia and intra-abdominal infections, until ESBLs started compromising usefulness in response to overuse and selective pressure. Organisms that produce ESBLs are an important reason for therapy failure with cephalosporins and have serious consequences for infection control. Furthermore, CTX-M ESBL enzymes have been associated with coresistance to other agents including trimethoprim-sulphamethoxazole, tetracycline, gentamicin, tobramycin and ciprofloxacin.^5^ It is essential that clinical microbiology laboratories rapidly and reliably detect and report ESBL-producing organisms.

## Case report

An eight-year-old girl presented with a two-week history of fever, abdominal pain, passage of watery stool and recurrent vomiting. There was also a history of frequent micturition with occasional dysuria but neither haematuria nor passage of dark-coloured urine. Prior to presentation in the teaching hospital, she had been admitted to a distant private hospital for five days, where she was treated with amoxicillin, ciprofloxacin and artesunate (doses and duration of treatment are unknown). She was discharged from that hospital because her state of health was not improving significantly and also because of the need for better family support. There was no other history of hospital admission or blood transfusion and no history suggestive of haemoglobinopathy. Immunisation and nutritional history were essentially normal.

General physical examination revealed a conscious but ill-looking, somewhat pale, febrile (temperature 38.5 °C) girl. She was moderately dehydrated and jaundiced. Her weight was 22 kg (86% of expected weight for age). There was no facial or pedal oedema. The respiratory rate was 32 cycles per min and breathing was regular; pulse regular at 120 beats per min and with good volume. Her blood pressure was 90/50 mmHg. The significant systemic findings on examination at admission were severe suprapubic tenderness, moderate hepatosplenomegaly (firm, not tender) and negative renal angle tenderness. All other systems were normal.

Conventional blood cultures were done at five different times after admission. There was a growth of *Klebsiella* sp. on three occassions. Once, the isolate was sensitive to gentamicin and ceftriaxone but resistant to all available antibiotics tested on the other two occasions. Urine and stool cultures did not yield growth of any pathogens. Screening for human immunodeficiency virus (HIV), hepatitis C virus (HCV) and hepatitis B surface antigen (HBsAg) was negative.

Full blood counts showed a haematocrit ranged between 14% and 30%, white cell counts of 8 x 10^9^/L – 9.2 x 10^9^/L and an essentially normal platelet count (260 x 10^9^/L). The erythrocyte sedimentation rate was 80 mm/hr (Westergreen method) and the haemoglobin phenotype (by electrophoresis) was AS. Serum biochemistry parameters were all normal except for conjugated hyperbilirubinaemia. Repeated abdominal ultrasonography showed findings that are consistent with hepatosplenomegaly in a septicaemic patient.

Whilst in hospital and when fever was uncontrolled and persistent, the patient was given fresh whole blood transfusions thrice and exchange blood transfusions twice, amongst other forms of treatment.

Fever remained persistent for five weeks following admission, despite different courses of antibiotics involving ciprofloxacin, gentamicin, ceftazidime, ceftriaxone and amoxicillin-clavulanic acid (Table 1). It was only at this point that the possibility of infection with an ESBL-producing organism was considered. ESBL-producers are not routinely sought in the diagnostic laboratory. During the sixth week, the *Klebsiella* sp. isolate from the patient was tested and confirmed to be producing an ESBL. Immediately following this test result, treatment was commenced with imipenem (not routinely available in the hospital) and there was dramatic resolution of fever. The patient remained free of fever for one week after receiving imipenem and was subsequently discharged. Two weeks later, when she reported for follow-up, she was still fever-free and healthy.

**TABLE 1:** Treatment interventions.

S/No.	Period	Treatment	Cost	
			NGN	USD
1	First week	I/V ciprofloxacin & I/M gentamicin	3780.00	24.55
2	Second week	I/V ceftazidime & I/M gentamicin	10 920.00	70.91
3	Third/Fourth week	I/V ceftriaxone & I/M gentamicin	8500.00	55.19
4	Fifth week	I/V amoxicillin/clavulanate & I/M gentamicin	6800.00	44.16
5	Sixth week	I/V imipenem/cilastatin	50 400.00	327.27
6	-	Blood transfusions	9500.00	61.69
	**Total cost**		**89900.00**	**583.77**

S/No., Serial number; NGN, Nigerian Naira; USD, United States Dollar; I/V, intravenous; I/M, intramuscular.

The *Klebsiella* sp. isolate was identified as *Klebsiella pneumoniae* subspecies *pneumoniae* using the API 20E identification strips for Enterobacteriaceae (bioMérieux, Marcy-l’Étoile, France). Presumptive ESBL phenotypic testing and confirmation in the organism was performed by disc diffusion tests on Mueller Hinton agar by employing ceftazidime (30 µg) and cefpodoxime (10 µg) alone and in combination with clavulanic acid as ceftazidime-clavulanic acid (30/10 µg) and cefpodoxime-clavulanic acid (10/1 µg) respectively. Results were interpreted using the Clinical and Laboratory Standards Institute (CLSI) criteria for disc diffusion.^6^ Antimicrobial susceptibility testing for the organism was carried out by the disc diffusion technique according to the guidelines and recommendations of CLSI.^6^ The isolate was resistant to streptomycin, gentamicin, chloramphenicol, tetracycline, nalidixic acid, ciprofloxacin, ampicillin, trimethoprim, sulphamethoxazole, ceftriaxone, cefepime and amoxicillin-clavulanic acid, but susceptible to imipenem.

**FIGURE 1:**
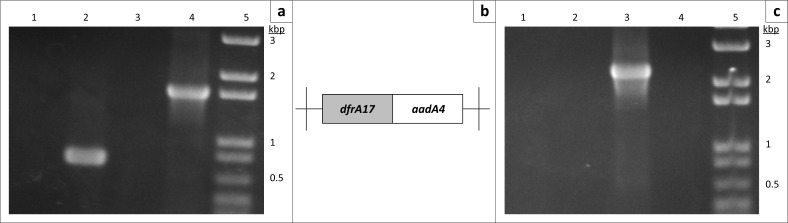
PCR amplification of the variable regions of class 1 and class 2 integrons. (a) Class 1 integron-variable regions amplified using Lev5’CS and Lev3’CS primers. Lane 1: No template; Lane 2: *E. coli* strain 042 bearing an *aadA* cassette within a class 1 integron; Lane 3: *E. coli* strain 17-2 bearing the *dfrA1-sat-aadA* cassette sequence within a class 2 integron; Lane 4: *K. pneumoniae* subsp. *pneumoniae* isolate K01 from this study; Lane 5: 1 kb Ladder plus (Invitrogen). Marker size fragments are indicated to the right of the gel in kilobase pairs. (b) Class 2 integron-variable regions amplified using hep51 and hep74 primers with samples shown in (a) loaded on to the gel. (c) Cassette content and orientation of the K01 integron amplified in (A), as predicted from the DNA sequence.

**FIGURE 2:**
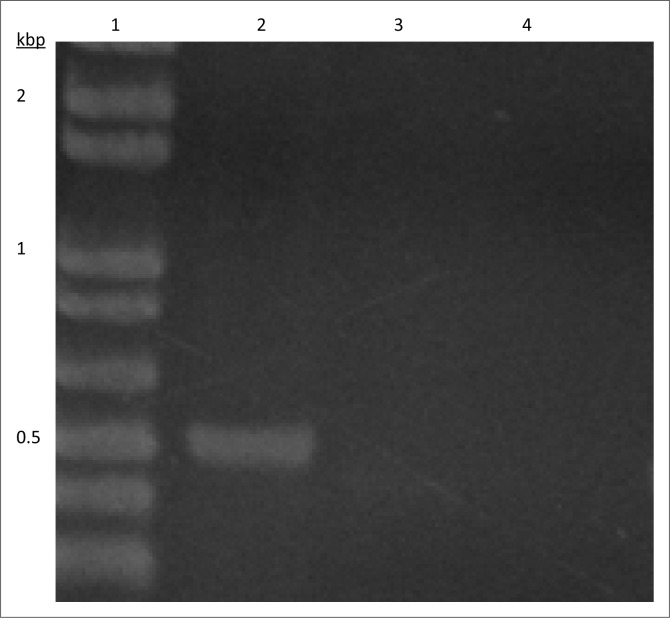
PCR amplification of a ctx allele from *K. pneumoniae* subsp. *pneumoniae* isolate K01 from this study. Lane 1: 1 kb Ladder plus (Invitrogen). Lane 2: K01. Lane 3: ESBL-negative *E. coli* strain 042. Lane 4: No template. Marker size fragments are indicated to the left of the gel in Kilobase pairs. In the absence of a positive control, the identity of the band amplified from strain K01 was determined by sequencing.

Genomic DNA was extracted from the isolate using the Wizard genomic extraction kit (Promega) according to the manufacturer’s directions and used as template for PCR reactions targeting resistance elements and genes. Platinum PCR Supermix (Invitrogen) was used for all reactions, and PCR cycle conditions were as recorded in the original articles describing the primers (Table 2). We employed oligonucleotides that prime the conserved ends of the cassette regions of class 1 and 2 integrons respectively to screen for these elements (Table 2).^7,8^ As shown in Figure 1, we were able to determine that the strain harboured a class 1, but not a class 2 integron. Sequencing of the 1.6 kb amplified class 1 cassette region revealed that it was identical to the cassette region of plasmid pIP1206 (Genbank Accession number NC_010558), containing two integrated cassettes: a *dfrA17* cassette encoding resistance to trimethoprim, and an *aadA4* aminoglycoside resistance cassette.^9^ Since the integron did not contain an ESBL cassette, we screened the isolate for *bla*_CTX-M_ type genes, employing primers that amplify an internal fragment from multiple *bla*_CTX-M_ alleles (Table 2)^10^. The resulting 550 bp product shown in Figure 2 was sequenced and found to be identical to the corresponding region of *bla*_CTX-M-15_.

**TABLE 2:** Oligonucleotides for PCR reactions.

Target gene	Primers		Amplicon size	Reference
	Name	Sequence		
Class 1 integron cassette region	Lev5’CS	5’-GGC ATC CAA GCA GCA AG-3’	Varies with cassette content (0.7 Kb for *aadA* in control strain 042)	7 (Lévesque et al.)
	Lev3’CS	5’ AAG CAG ACT TGA CCT GA-3’		
Class 2 integron cassette region	hep74	5′- CGG GAT CCC GGA CGG CAT GCA CGA TTT GTA- 3′	Varies with cassette content (2.2 Kb for *dfrA1-sat1-aadA1* in control strain 17-2)	8 (White et al.)
	hep51	5′-GAT GCC ATC GCA AGT ACG AG-3′		
CTX-M genes	CTX-MA	5′-CGC TTT G CG ATG TGC AG-3′	0.55 Kb	10 (Bonnet et al.)
	CTX-MB	5′-ACC GCG ATA TCG TTG GT-3′		

Note: Please see the full reference list of the article, Aboderin AO, Adefehinti O, Odetoyin BW, Olotu AA, Okeke IN, Adeodu OO. Prolonged febrile illness due to CTX-M-15 extended-spectrum β-lactamase-producing *Klebsiella pneumoniae* infection in Nigeria. Afr J Lab Med. 2012;1(1), Art. #16, 4 pages. http://dx.doi.org/10.4102/ajlm.v1i1.16, for more information.

The cost of repeated investigations (Table 3) was N14 000.00 ($90.92), which is more than a tenfold increase on projected diagnostic expenses, had a diagnosis estimate been made immediately on admission. Antibiotics and fresh whole blood transfusions (thrice) as well as exchange blood transfusions (twice) cost N89 900.00 ($583.77). These and other treatment interventions effectively doubled the cost of treatment as compared to that for what would normally have been appropriate therapy after admission. Finally, prolonged hospital accommodation, feeding and nursing care over 48 days amounted to N20 400.00 ($132.46) in contrast to N2975.00 ($19.32) for admission for one week, an almost tenfold increase.

**TABLE 3:** Investigations.

Test	Cost	
	NGN	USD
Blood culture	1500.00	9.74
Screening for malarial parasites	525.00	3.41
Stool culture	300.00	1.95
Urine culture	250.00	1.62
Haematocrit estimation	3200.00	20.78
Full blood count, ESR	2250.00	14.61
Direct Coomb’s test	500.00	3.25
Haemoglobin phenotyping	300.00	1.95
HCV and HBV screening	2000.00	12.99
Chest X-ray	375.00	2.44
Abdominal ultrasound	1500.00	9.74
Plasma electrolyte and urea estimation	1100.00	7.14
Urinalysis	200.00	1.30
**Total**	**14 000.00**	**90.92**

## Discussion

The occurrence and spread of infections resulting from ESBL-producing organisms have been well documented in countries of Europe, Asia and North America.^11^ In contrast, data on the epidemiology of ESBL enzymes is very limited in Nigeria. Molecular analysis of eight Nigerian ESBL-producing *Enterobacter* species in 2001 detected only TEM and SHV-like ESBLs and no CTX-M types.^12^ In a study of *Klebsiella pneumoniae* isolates associated with community-acquired urinary tract infections between 2002 and 2003 in Ibadan, Nigeria, CTX-M group 1, -like enzymes were found in 17 (57%), but CTX-M-15 was identified in only two isolates.^13^ Olowe et al. investigated the occurrence of CTX-M ESBL-producing *E. coli* and found nine of 79 ampicillin-resistant hospital isolates to be ESBL producers.^14^ More recently, a case of necrotising fasciitis was reported in a Nigerian patient in the UK.^15^ *Morganella morganii* and *Citrobacter freundii* carrying the CTX-M-15 ESBL gene were isolated from the patient, highlighting the presence of CTX-M genes in Africa even though there is a scarcity of reports in the literature. Here, we describe a case of prolonged, uncontrolled fever found to be due to ESBL-producing *K. pneumoniae*. To the best of our knowledge, this is the first documented clinical course and outcome of ESBL-producing bacterial infection in Nigeria.

Failure to recognise and initially diagnose the presence of an ESBL-producing organism resulted in considerable expense in the management of the infection. This includes the cost of different courses of ineffective antibiotics for five weeks, exchange blood transfusions and whole blood transfusions, as well as hospital charges resulting from prolonged stay in hospital. The estimated avoidable cost of supportive therapy and investigation related to possible alternative diagnoses was almost $600 in a country where the average annual per capita income is $2300 and health care resources are severely limited.

The clinical diagnostic microbiology laboratory plays a crucial part in the detection and reporting of ESBL-producing bacteria, and it is important that laboratories be fully aware of the significance of ESBL-producing organisms and the best methods for detecting them, as in our case. Resource-limited hospitals can incur prohibitive costs associated with ESBL-producer infections because of prolonged supportive therapy and treatment failure following the use of readily available antibiotics. Diagnostic improvements to allow routine detection and reporting of ESBL production in Enterobacteriaceae will help greatly in avoiding these costs.
